# Avian Cerebellar Floccular Fossa Size Is Not a Proxy for Flying Ability in Birds

**DOI:** 10.1371/journal.pone.0067176

**Published:** 2013-06-25

**Authors:** Stig A. Walsh, Andrew N. Iwaniuk, Monja A. Knoll, Estelle Bourdon, Paul M. Barrett, Angela C. Milner, Robert L. Nudds, Richard L. Abel, Patricia Dello Sterpaio

**Affiliations:** 1 Department of Natural Sciences, National Museums Scotland, Edinburgh, United Kingdom; 2 Canadian Centre for Behavioural Neuroscience, University of Lethbridge, Lethbridge, Alberta, Canada; 3 School of Social Sciences, University of the West of Scotland, Paisley, United Kingdom; 4 Department of Earth Sciences, The Natural History Museum, London, United Kingdom; 5 Faculty of Life Sciences, University of Manchester, Manchester, United Kingdom; 6 Faculty of Medicine, Imperial College London, London, United Kingdom; 7 School of Engineering, Computing and Applied Mathematics, University of Abertay, Dundee, United Kingdom; University of Alberta, Canada

## Abstract

Extinct animal behavior has often been inferred from qualitative assessments of relative brain region size in fossil endocranial casts. For instance, flight capability in pterosaurs and early birds has been inferred from the relative size of the cerebellar flocculus, which in life protrudes from the lateral surface of the cerebellum. A primary role of the flocculus is to integrate sensory information about head rotation and translation to stabilize visual gaze via the vestibulo-occular reflex (VOR). Because gaze stabilization is a critical aspect of flight, some authors have suggested that the flocculus is enlarged in flying species. Whether this can be further extended to a floccular expansion in highly maneuverable flying species or floccular reduction in flightless species is unknown. Here, we used micro computed-tomography to reconstruct “virtual” endocranial casts of 60 extant bird species, to extract the same level of anatomical information offered by fossils. Volumes of the floccular fossa and entire brain cavity were measured and these values correlated with four indices of flying behavior. Although a weak positive relationship was found between floccular fossa size and brachial index, no significant relationship was found between floccular fossa size and any other flight mode classification. These findings could be the result of the bony endocranium inaccurately reflecting the size of the neural flocculus, but might also reflect the importance of the flocculus for all modes of locomotion in birds. We therefore conclude that the relative size of the flocculus of endocranial casts is an unreliable predictor of locomotor behavior in extinct birds, and probably also pterosaurs and non-avian dinosaurs.

## Introduction

Paleoneurology investigates the evolution of the vertebrate brain through time and makes inferences about the behavior of extinct vertebrates using two main sources of information. The first uses the morphology of the internal surface of the brain cavity in fossil skulls, whether revealed through damage (pre- or post-preservational), sectioning or as natural, synthetic or digital casts of the cavity. The second comes from advances in our understanding of behavior-related neural function in extant animals. However, this information is only useful if differences in neural function are causally related to changes in brain region volume that are expressed in the external morphology of the brain, and if the impression of the brain on the internal surface of the brain cavity is reasonably accurate [1]. While comparative neurology has advanced greatly over the last century [2], arguably the most important improvement in paleoneurological investigation has been the advent of non-invasive X-ray tube and synchrotron source micro computed-tomography (µCT) imaging [3]. Although these techniques are affected by similar problems to those inherent in older serial sectioning methods [4], they have greatly increased the total number of fossil taxa for which endocranial anatomy is known [3].

The degree to which the brain actually fills the brain cavity is known to vary greatly across vertebrate clades [1]. However, unlike their nearest living relatives, the crocodiles, brain cavity volume in birds is broadly comparable to that of the brain it houses [5]. Hence, casts of the avian brain cavity represent reasonable approximations of external brain morphology, and thus the endocranium of bird fossils can provide insights into the correlated evolution of the brain and flight. Based on current evidence, it appears that the avian brain, at least in some taxa, was already fully modern in form and relative size by 55 Mya [6]. However, full or partial brain morphology is known only for a few Mesozoic bird species, and elucidating the timing of these changes has been frustrated by an absence of suitable fossils. Those that are known, particularly that of the ‘London’ specimen of the Late Jurassic *Archaeopteryx lithographica* [7], do indicate enlargement (relative to a putative ancestral crocodile-like condition) of the telencephalon, mesencephalon and cerebellum, including a pronounced outgrowth of the cerebellar flocculus [8].

The suite of neuroanatomical changes observed in *Archaeopteryx* is generally assumed to relate to enhancement of somatosensory control during the evolution of flight [7]. This assumption is supported by the observation that the other archosaurs to have evolved powered flight, pterosaurs, also had regional expansions similar to those of birds, even though their overall brain size was not necessarily as great [9]. Several bird-like theropod dinosaurs also possessed brains with an avian-like morphology closer to that of living birds than to the brain morphology of *Archaeopteryx* [10]–[14]. One possible reason is that these bird-like theropods are secondarily flightless birds [15], [16], although this explanation has generally been rejected on the basis of strong phylogenetic evidence to the contrary [17], [18]. Another is that the occurrence of an avian-like brain in birds and some non-avian dinosaurs indicates that a ‘flight-ready’ brain was already present in the common ancestor of both groups [19], and possibly also pterosaurs. However, recognition of such a ‘flight-ready’ brain on the basis of external brain morphology, the only data available from fossils, is likely to prove problematic.

One feature that is easily visible on endocranial casts and that offers some potential for use as an indicator of neural flight control is the cerebellar flocculus. The flocculus is involved in adaptive processing of two important reflexes: the vestibulo-collic reflex (VCR), which acts to stabilize the head through cervical musculature, and the vestibulo-ocular reflex (VOR), which acts to maintain a stable image on the retina during rotational head movements. The VOR works by integrating information from the vestibular and visual systems to generate compensatory motor impulses to the extraocular muscles such that the eyes are automatically moved opposite to the direction of head rotation [20]. VOR gain will differ depending on changes in optic flow between environments, so VOR processing must be plastic and adaptive to reduce error and respond rapidly to changing circumstances, such as alterations in flight speed, landing and terrestrial locomotion [21].

Avoiding retinal image slip is obviously crucial for flight, particularly in tight complex environments with fast optic flow fields such as forests where collisions are likely, or where flight becomes unstable due to low ground speeds. Consequently, species engaging in fast and complex aerial maneuvers (e.g., aerial predators) or unstable low speed or hovering flight (e.g., hummingbirds) are likely to have a greater requirement for accurate visual field and head stabilization. Since a greater proportion of neural mass must be dedicated to VOR/VCR processing in these species, the flocculus would be assumed to be larger in those than in species that usually fly in open environments with relatively simple, slow moving visual flow fields (e.g., far-field horizon), or are fully flightless. Indeed, hummingbirds have undergone an expansion of the pretectal nucleus lentiformis mesencephali [22], which is involved in the optokinetic response and projects to the flocculus [23]. Whether a similar expansion has occurred in the flocculus of hummingbirds and other highly maneuverable fliers has not been tested, but a prediction based on previous paleoneurological studies is that the flocculus would be proportionately larger in acrobatic fliers than in poor fliers or flightless birds.

In extinct taxa, this prediction has led to the size of the flocculus relative to the rest of the endocranial cast being used to infer flight capability in fossil birds [6], [7], [24] and pterosaurs [9]. However, in a paleoneurological context this prediction actually relates to floccular fossa size, and in life this structure may also house significant amounts of vasculature. For example, a floccular sinus and rostral and caudal floccular arteries are normally present and contiguous with the neural flocculus [25], but their boundaries in the fossa can be difficult to determine. The relative contribution of vasculature to the volume of the floccular fossa is presently unclear in extant birds and unknown in extinct avian taxa, because variation in the size and development of these structures has not been surveyed across extant avian clades. The presence of these vascular structures may lead to an overestimation of neural flocculus size in endocranial casts. Conversely, portions of the floccular lobes situated within the body of the cerebellum cannot be determined from endocranial casts, so floccular fossa casts may underestimate true neural flocculus volume. These uncertainties undermine the reliability of using relative ‘flocculus’ size on avian endocranial casts to infer locomotor capability in extinct birds, non-avian dinosaurs and pterosaurs. However, the existence of a reliable relationship between floccular fossa size and flying ability has never been tested.

Here, we address this issue by using µCT to reconstruct digital casts of the brain cavity in extant bird species with known locomotor behavior, in order to test correlations between relative ‘flocculus’ size (as indexed by the relative volume of the floccular fossa) and flying ability.

## Materials and Methods

Sixty avian species (Table 1) were selected for scanning from the collections of National Museums Scotland, Edinburgh (NMS) and The Natural History Museum, London (NHMUK) based on known flying ability. All selected species are extant except for the Rodrigues Solitaire (*Pezophaps solitarius*). X-ray µCT Scanning was performed between 12 µm and 149 µm voxel size (mean = 56 µm, s.d. = 24.7 µm) using HMXST CT systems [26] at NHMUK, University of Abertay, Dundee and Nikon Metrology, Tring. Detailed information about dataset species composition, flight style categorization and scanning parameters can be found in Scanning S1. The study used only museum specimens of skulls, so no permits were required for the described study, which complied with all relevant regulations.

**Table 1:** Taxa used in this study, including volume measurements and floccular fossa morphology.

Order	Genus and Species		BCEV (mm^3^)		FFV (mm^3^)	% of BCEV	Fossa Type
Tinaniformes	*Rhynchotus rufescens*		3690.58		14.86	0.40	Type 3
Apterygiformes	*Apteryx haastii*		12496.13		32.24	0.26	Type 3
Struthioniformes	*Casuarius casuarius*		32724.27		258.47	0.79	Type 3
Struthioniformes	*Struthio camelus*		36517.99		195.92	0.54	Type 3
Struthioniformes	*Dromaius novaehollandiae*		27054.50		236.13	0.87	Type 3
Rheiformes	*Rhea americana*		13713.05		153.76	1.12	Type 3
Anseriformes	*Aythya fuligula*		5351.00		38.97	0.73	Type 2
Anseriformes	*Cygnus olor*		17360.36		149.53	0.86	Type 5
Anseriformes	*Tachyeres brachypterus*		6667.40		92.15	1.38	Type 5
Galliformes	*Gallus gallus*		3976.07		35.87	0.90	Type 5
Galliformes	*Phasianus colchicus*		4021.23		29.78	0.74	Type 5
Gruiformes	*Grus grus*	19959.78			166.06	0.83	Type 5
Gaviiformes	*Gavia immer*		12284.93		179.58	1.46	Type 5
Podicipediformes	*Podiceps cristatus*		3303.11		44.61	1.35	Type 5
Sphenisciformes	*Eudyptula* sp.		8522.17		64.30	0.75	Type 2
Procellariiformes	*Diomedea exulans*		29151.60		192.40	0.66	Type 3
Procellariiformes	*Pelagodroma marina*		496.91		3.92	0.79	Type 2
Procellariiformes	*Fulmarus glacialis*		7440.16		48.96	0.66	Type 5
Procellariiformes	*Pelecanoides urinatrix*		1351.72		21.11	1.56	Type 2
Pelecaniformes	*Pelecanus erythrorhynchos*		13012.42		105.12	0.81	Type 5
Pelecaniformes’	*Fregata magnificens*		10389.53		53.67	0.52	Type 2
Pelecaniformes’	*Phalacrocorax carbo*		13440.04		82.20	0.61	Type 5
Pelecaniformes’	*Phalacrocorax harrisi*		10936.73		71.37	0.65	Type 5
Pelecaniformes’	*Threskiornis aethiopicus*		9643.49		54.17	0.56	Type 5
Phaethontiformes	*Phaethon lepturus*		2801.90		38.81	1.39	Type 4
Ciconiiformes	*Ciconia ciconia*		11348.13		56.15	0.49	Type 5
Ciconiiformes	*Ardea cinerea*		4999.82		69.61	1.39	Type 5
Charadriiformes	*Rhynchops niger*		1235.81		8.43	0.68	Type 5
Charadriiformes	*Larus argentatus*		5716.45		26.94	0.47	Type 4
Charadriiformes	*Creagrus furcatus*		4919.36		16.62	0.34	Type 4
Charadriiformes	*Gelochelidon nilotica*		1900.16		17.36	0.91	Type 5
Charadriiformes	*Stercorarius skua*		6769.41		38.13	0.56	Type 5
Charadriiformes	*Alca torda*	3285.72			47.04	1.43	Type 5
Strigiformes	*Tyto alba*	6521.50			21.49	0.33	Type 5
Falconiformes	*Buteo buteo*		7851.35		33.22	0.42	Type 5
Falconiformes	*Aquila chrysaetos*		21045.03		104.74	0.50	Type 5
Falconiformes	*Circus cyaneus*		3928.72		25.86	0.66	Type 2
Falconiformes	*Vultur gryphus*		27099.93		383.23	1.41	Type 5
Falconiformes	*Sagittarius serpentarius*		12912.27		124.33	0.96	Type 5
Falconiformes	*Falco tinnunculus*		3152.49		18.01	0.57	Type 4
Falconiformes	*Falco subbuteo*		2989.74		13.66	0.46	Type 4
Falconiformes	*Pandion haliaetus*		10146.91		76.46	0.75	Type 5
Opisthocomiformes	*Opisthocomus hoatzin*		3370.00		33.33	0.99	Type 5
Psittaciformes	*Ara macao*	15157.87			29.08	0.19	Type 1
Psittaciformes	*Amazona aestiva*		8511.51		35.24	0.41	Type 1
Psittaciformes	*Strigops habroptila*		8849.56		26.06	0.29	Type 1
Columbiformes	*Columba livia*		2134.52		14.84	0.70	Type 2
Columbiformes	*Pezophaps solitaria*		8665.89		48.72	0.56	Type 5
Caprimulgiformes	*Podargus strigoides*		2322.97		14.84	0.64	Type 4
Caprimulgiformes	*Steatornis caripensis*		2039.77		14.55	0.71	Type 5
Apodiformes	*Apus apus*	707.83			5.25	0.74	Type 4
Apodiformes	*Selasphorus rufus*		157.29		1.64	1.04	Type 1
Trogoniformes	*Trogon curucui*		889.99		5.59	0.63	Type 4
Coraciiformes	*Alcedo atthis*		741.51		8.12	1.10	Type 5
Coraciiformes	*Coracias garrulus*		1970.03		10.69	0.54	Type 4
Piciformes	*Ramphastos dicolorus*		4525.02		34.45	0.76	Type 2
Passeriformes	*Tyrannus tyrannus*		532.71		1.18	0.22	Type 4
Passeriformes	*Hirundo rustica*		217.36		4.08	1.88	Type 4
Passeriformes	*Corvus corax*		17924.59		78.04	0.44	Type 4
Passeriformes	*Acanthorhynchus superciliosus*		2369.64		28.89	1.22	Type 2

Digital endocranial casts were created using the Livewire interpolation and localized threshold segmentation tools in Materialise Mimics 14.11 by S.A.W. and M.A.K. Vascular features (e.g., occipital sinus, semicircular veins) on the endocranial surface were retained partly to maintain consistency with earlier quantitative studies that explored endocranial volume by particle-fill or fluid displacement methods [1], and partly because their removal is highly problematic as it involves fundamental uncertainties concerning the boundary between the vascular features and neural tissue in life [27]. However, where major vascular foramina that extend from the brain cavity to the exterior of the skull (e.g., foramina of the paired carotid arteries and caudal sections of the semicircular veins) have well-defined junctions with the neural endocranial cast, these were removed using the 3D voxel editing tool in Mimics 14.11, which allows the removal contour to follow the curve of the surface of the main endocranial cast. The rostral portion of the semicircular vein between the cerebellar fossa and mesencephalic fossa varies between being a fully enclosed canal (e.g., *Columba livia*, *Corvus corax*) and a sulcus (e.g., *Muscivora tyrannus*, *Podiceps cristatus*) in this dataset, so this portion of the structure was left intact in all segmentations to maintain consistency among brain cavity casts. Cranial nerves were segmented along the length of their foramina up to their exit from the brain cavity wall. The nerves were included in the brain cavity volume measurements because their diameter should broadly relate to the thickness of each nerve bundle and thus the relative importance of sensory and motor projections to relevant processing centers in the brain [28].

**Figure 1:**
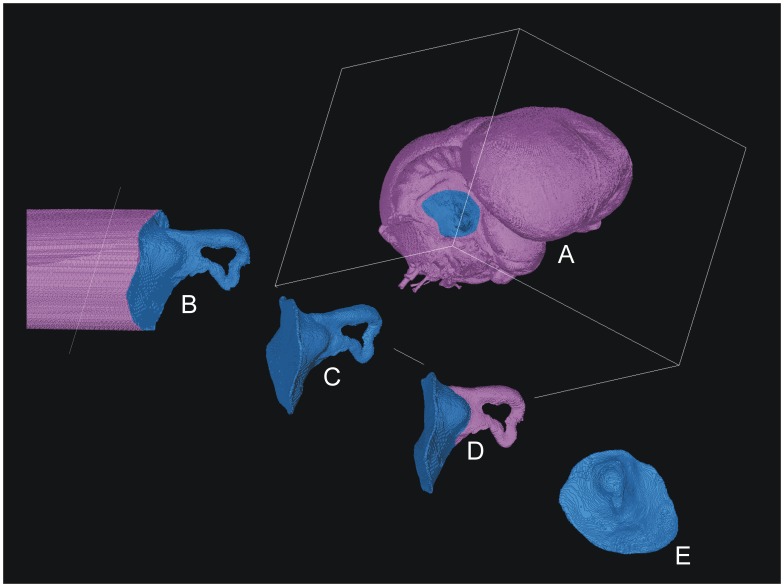
Segmentation process for the floccular fossa endocast (*Corvus corax*) using the Materialise Mimics 14.11 3D editing tool. **A**. a separation contour is chosen on the segmented endocranial cast model based on assessment of which contour best represents the point at which the lateral wall of the cerebellar fossa most sharply projects laterally to form the walls of the floccular fossa. Voxels surrounding the separation contour are selected and deleted. **B**. voxels medial of the separation contour are removed following the curve of the contour, which normally results in a concave medial surface (**C**.). **D**. vascular structures are removed from the FFV endocast where required (Type 4 fossa shown in this example) to leave (**E**.) only the external expression of the fossa as an endocast.

The floccular fossa casts were separated from the digital endocranial casts using the Mimics 14.11 3D voxel editing tool. Unlike polygon mesh editing approaches [6], [29], this technique results in precise orthogonal divisions between voxel boundaries that can be refit with no distortion or loss of information. On all endocranial casts, the separation between the floccular fossa and endocranial cast was made at the contour that best marks a sharp change of angle between the lateral wall of the cerebellar fossa and the floccular fossa proper. Remaining voxels projecting medially from the junction were removed, resulting in a contoured surface following the separation contour (Figure 1).

The floccular fossa was morphologically variable in this dataset and the true extent of the neural flocculus in the fossa was often difficult to determine from the bony walls of the floccular fossa alone. Separating the parts of the fossa assumed to be associated with vascular structures from those presumably containing neural tissue would be a highly subjective process. Because previous inferences of flying behavior have been made based on assessment of the size of the entire fossa [6], [7], [9], [24], no attempt was made to separate these structures, although vascular foramina exiting the fossa distally (e.g., rostral and caudal floccular arteries) were removed from the floccular fossa endocast.

Volume measurements (mm^3^; Data S1) were made by S.A.W. from voxel data using Mimics 14.11. These comprised measurements of the full brain cavity endocranial casts with the floccular fossa included (BCEV) and the separated left and right floccular fossa casts, which were combined as a single volumetric value (FFV). FFV values were subtracted from the BCEV values resulting in a reduced brain cavity cast (BCEVr), and both FFV and BCEVr measurements were Log_10_ transformed to normalize the data and mitigate size effects within the dataset.

To examine interspecific differences in relative FFV size, we ran an ordinary least-squares linear regression on FFV and BCEVr. Using species as independent data points, we calculated residuals from this regression, which were used as relative FFV values in the analyses of aerial maneuverability described below. In addition, we calculated a phylogeny-corrected linear regression and prediction intervals to assess whether any of the species were significant outliers [30]–[32]. Two different phylogenetic trees were constructed in Mesquite [33]. The two trees differed in the branching of deeper nodes in the phylogeny (e.g., orders, families) and were based on Hackett et al. [34] and Livezey and Zusi [35]. Additional resolution within clades was provided by Kennedy and Page [36] and Harshman et al. [37]. Because the trees were assembled from multiple sources, all branch lengths were set to 1 to calculate the phylogeny-corrected regression line and prediction intervals. Once the regression line and prediction intervals were calculated in the PDAP module [38] of Mesquite, they were re-plotted in the original data space (following [30]).

The relationship between the two transformed volume values and flight was tested using four published indices of aerial maneuverability derived from wing bone proportions (Data S1). The first of these, the brachial index (BI) [39], comprises humerus to ulna length ratios that represent a continuum in which maneuverable species possess low values (0.7 or lower), poorly maneuverable gliding and soaring species have values of 1.0 or higher, and flightless species have high values of 1.2 or higher. Two published analyses by Rayner [40] and Norberg [41] of flight style categories based on wing loading and aspect ratios were also tested. Categories from these studies were coded and in some cases combined to emphasize aerial maneuverability. An extra ‘flightless’ category was also added. The Rayner [40], categories comprised (0) flightless; (1) poor fliers; (2) generalists occupying non-specialized positions; (3) marine and thermal soarers; (4) diving and water birds and (5) aerial predators. The Norberg [41] categories were (0) flightless; (1) slow, poorly maneuverable soarers; (2) fast, poorly maneuverable fliers; (3) slow maneuverable fliers; (4) fast maneuverable fliers. Lastly, the wing bone proportion/kinematic categories of Wang et al. [42] were included, but reordered to reflect maneuverability: (0) flightless; (1) flapping and soaring (comparable to Rayner [40], category 3, and Norberg [41] categories1 and 2); (2) continuous flapping (comparable to Rayner [40], categories 1 and 4, and Norberg [41] category 2); (3) bounding passerine-type flight (comparable to Rayner [40], category 2), and (4) flapping and gliding (comparable to Rayner [40], category 5, and Norberg [41], categories 3 and 4). A comparison between relative floccular fossa volume in volant versus non-volant taxa was also made.

Each of these indices of aerial maneuverability was then compared with relative FFV (see above) using species as independent data points and phylogenetic generalized least-squares (PGLS), which takes phylogenetic relatedness into account [43]. Distance matrices and species data were exported from Mesquite and PGLS calculations performed in Regressionv2.m in MATLAB [43]. As with the phylogeny-corrected confidence intervals, all branch lengths were set at 1.

## Results

**Figure 2:**
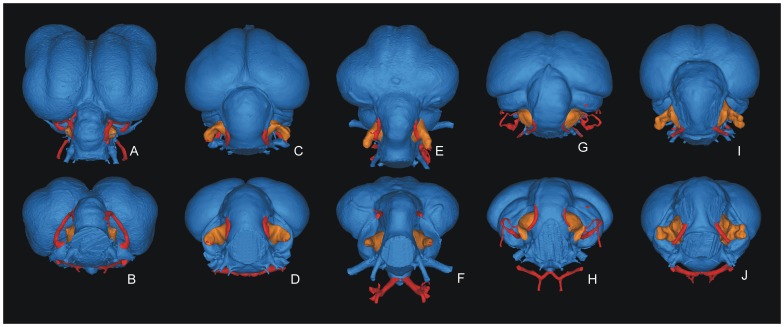
Flocculus types recognised in this study. A–B, Type 1 (*Ara macao*); C–D, Type 2 (*Eudyptula* sp.); E–F, Type 3 (*Struthio camelus*); G–H, Type 4 (*Apus apus*) and I–J, Type 5 (*Ardea cinerea*). Figures in the top row are dorsal views, figures in the bottom row are caudal views.

We detected five main morphological floccular fossa types in this dataset based on the degree to which the fossa is expanded within the loop of the rostral and caudal arteries, the shape of the proximal region of the fossa, the elongation of the fossa and its degree of rostrocaudal compression (Table 1). In Type 1 fossae (6.6% of sample; Figure 2 A,B) the arterial loop is enclosed within the fossa to the extent that the structures do not leave an impression on the fossa walls. The fossa itself is dome-shaped and only a single foramen exits the fossa distally. The arterial loop is also enclosed by Type 2 fossae (15% of sample; Figure 2 C,D), and the fossa base is dome-shaped and tapers distally into a rostrocaudally compressed region that twists to form a partial spiral. The fossa may be elongate or truncated, and its distal portion tapers into a single foramen that exits the fossa distally. Type 3 fossae (11.7% of sample; Figure 2 E,F) also enclose the arterial loop, but unlike Types 1 and 2, the base is not markedly domed, and exhibits no torsion. The main section of the fossa is elongate and approximately circular in section, either tapering into a single foramen that exits the distal extent of the fossa, or widening into a blunt and bulbous distal end. Type 4 fossae (20% of the sample; Figure 2 G,H) possess the same twisted base and rostrocaudal compression as Type 2 fossae, but do not enclose the arterial loop. In these the rostral and caudal arteries exit the tapered distal extent of the fossa and converge distally to form a ‘paperclip’ shape, with a single smaller distally-directed foramen at its distal extent. Finally, Type 5 fossae (46.7% of the sample; Figure 2 I,J) are the most variable. These lack the twisted base of Types 2 and 4, but are rostrocaudally compressed. The arterial loop leaves a distinct trace on the surface of the fossa, and there is often a sheet of bone in between that causes a ‘fenestra’ in the flocculus endocast. Variability in the development of the arterial sulci or foramina in these fossae may obscure the distal extent of the neural flocculus.

*Tyrannus tyrannus* possessed the smallest absolute FFV value (1.18 mm^3^) and *Vultur gryphus* the largest (383.23 mm^3^; Table 1). However, when expressed as a percentage of total BCEV, *Ara macao* possessed the smallest relative FFV (0.19%), while the relative FFV of *Hirundo rustica* was the largest (1.88%; Table 1).

**Figure 3:**
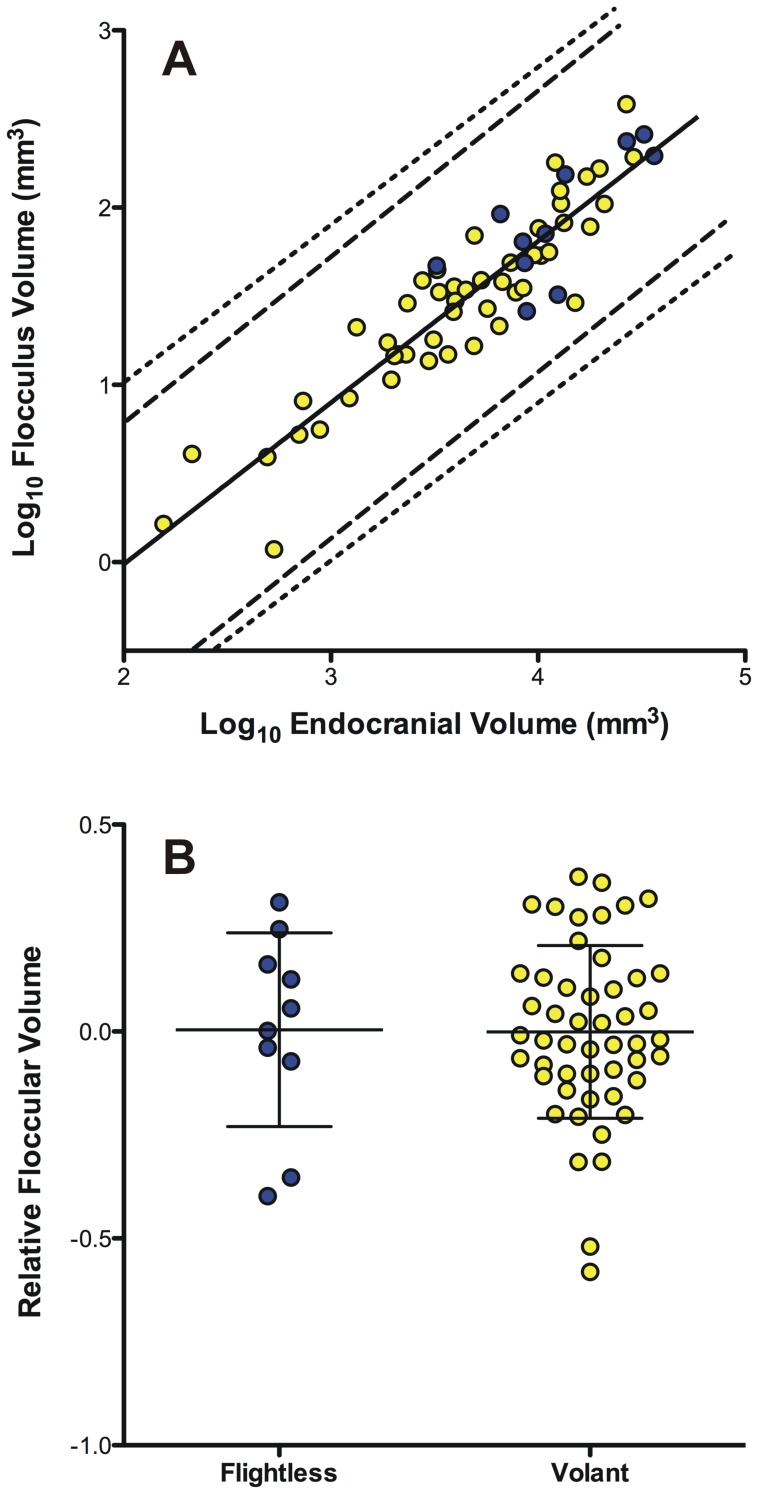
Floccular fossa volume relative to endocranial volume. **A**. scatterplot of floccular fossa volume plotted against total endocranial volume (minus that of the floccular fossa). The blue circles indicate flightless species whereas the yellow circles indicate volant species. The lines depict the least-squares linear regression line (solid, y = 0.919x+2.013) and 95% confidence intervals of least-squares linear regression using species as independent data points (dashed lines) and after correction for phylogeny (dotted lines). **B**. scatterplot of the relative floccular volume of flightless (blue) and volant (yellow) species calculated as the residuals from a common least-squares linear regression.

There was a strong positive correlation (p = <0.001, r^2^ = 0.84) between BCEVr and FFV and none of the species fell outside of the confidence intervals (Figure 3A). Although flightless species had larger absolute FFV volumes, there was a large amount of overlap in relative FFV volume between flightless and volant species (Figure 3B) and no significant difference between flightless and volant species was detected (all p values >0.10). This non-significant result remained when the wing-propelled divers were excluded from the analysis (p>0.10). Similarly, a comparison within clades revealed no appreciable differences in relative FFV. For example, the flightless Kakapo (*Strigops habroptilus*) has a relative FFV between that of the Scarlet Macaw (*Ara macao*) and the Blue-fronted Amazon (*Amazona aestiva*). Thus, the loss of flight is not associated with a significant change in relative FFV.

**Figure 4:**
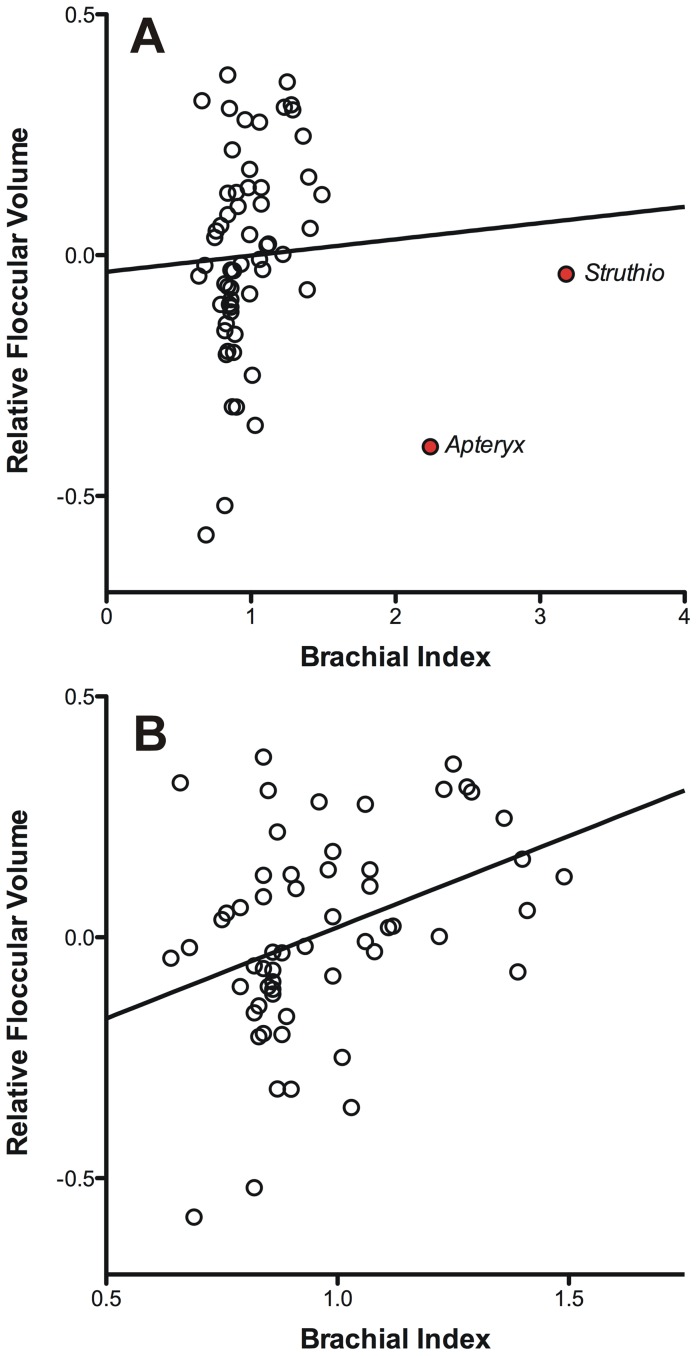
Scatterplot of relative floccular fossa volume plotted against the brachial index. **A**. including the outliers *Apteryx* and *Struthio*. **B**. with those outliers removed. Note that relative floccular fossa volume was calculated as the residuals from a least-squares linear regression as shown in Figure 3. For both scatterplots, the solid line depicts the least-squares linear regression.

The brachial index (BI) was not significantly associated with relative FFV in both our analyses of species as independent data points and PGLS (Table 2). As shown in Figure 4A, however, *Apteryx* and *Struthio* have far larger BI values (2.24 and 3.18 respectively) than all other taxa analyzed and are obvious outliers in the BI dataset. Excluding these two species from the analysis resulted in a significant positive relationship (Figure 4B) between relative FFV and BI, regardless of whether or not phylogeny was taken into account ([35]: F = 4.79, df = 1,55, p = 0.03, r^2^ = 0.08; [34]: F = 6.38, df = 1,55, p = 0.01, r^2^ = 0.10) or not (F = 8.82, df = 1,56, p = 0.004, r^2^ = 0.12).

**Table 2:** Results of analyses of variance on relative floccular volume and the three flight style estimates used in this study (see Materials and Methods for details).

Flight style estimate	No phylogeny	Livezey & Zusi [35]	Hackett et al. [34]
**Brachial index**	F = 0.22, df = 1,58, p = 0.64	F = 0.002, df = 1,57, p = 0.96	F = 0.05, df = 1,57, p = 0.82
**Rayner ** [**40**]	F = 2.19, df = 5,44, p = 0.07	F = 1.59, df = 5,44, p = 0.18	F = 1.33, df = 5,44, p = 0.27
**Norberg ** [**41**]	F = 2.03, df = 4,47, p = 0.11	F = 0.57, df = 4,47, p = 0.69	F = 0.76, df = 4,47, p = 0.56
**Wang et al.** [**42**]	F = 0.62, df = 4,27, p = 0.65	F = 0.09, df = 4,27, p = 0.98	F = 0.15, df = 4,27, p = 0.96

**Figure 5:**
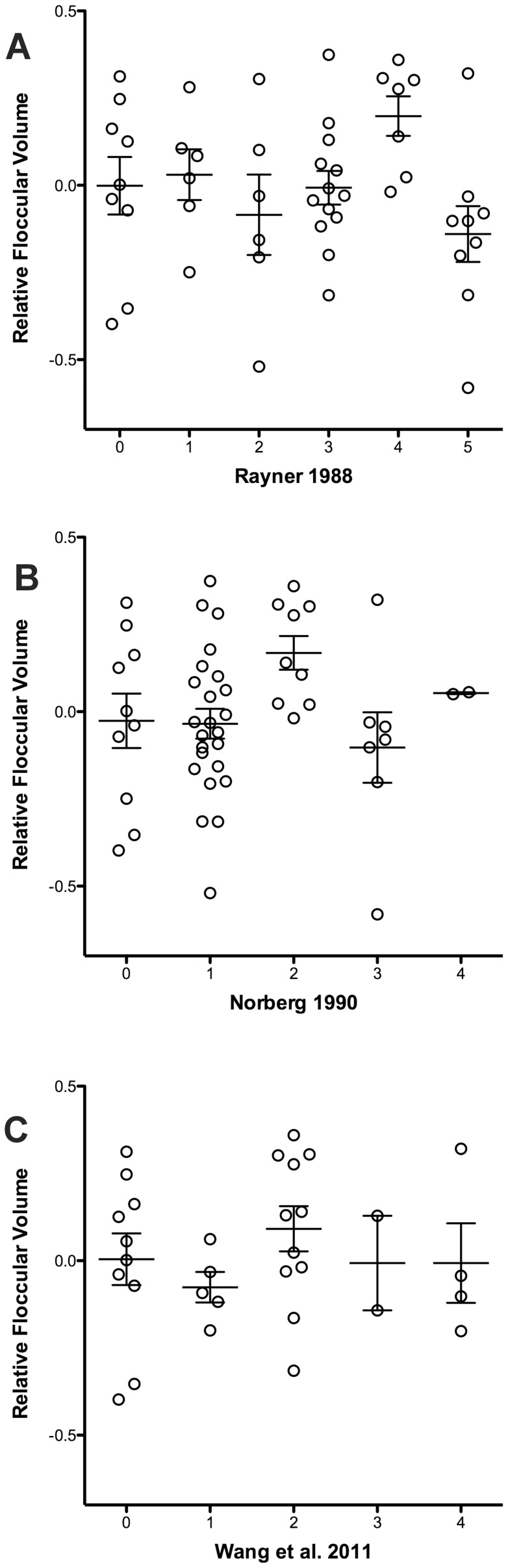
Scatterplots of relative floccular volume grouped according to each of the categories used in the aerial maneuverability measures. **A**. Rayner [40]. **B**. Norberg [41]. **C**. Wang et al. [42]. The mean ± standard deviations are shown for each group. Note that relative floccular volume was calculated as the residuals from a least-squares linear regression as shown in Figure 3.

Finally, no significant relationship was found between relative FFV and any of the three aerial maneuverability classification schemes (Figure 5, Table 2). Inspection of the scatterplots reveals that there is considerable variability within each category used in all three schemes and no clear difference in relative FFV among any of them.

## Discussion

Although there appears to be a relationship between relative FFV and BI, the need to remove the outliers *Apteryx* and *Struthio* to achieve significance even in this small selection of taxa indicates that relative floccular fossa size is not a reliable indicator of flying ability in extant birds. This finding is supported by the absence of a significant relationship between any categorical flight style variable and FFV, or even a clear separation between volant and flightless species.

Nonetheless, the weak relationship between relative FFV and BI is interesting as it suggests that a signal is present, but that it may have been weakened by one or more other factors. As mentioned above, it is possible that a significant proportion of the neural flocculus is not detected using this approach because it occurs within the vermis of the cerebellum and cannot be estimated from the endocranial surface. Nonetheless, current knowledge of flocculus extent within the avian cerebellum suggests that this is unlikely to be the case [44]. Another source of error may stem from determination of the demarcation between the floccular fossa and cerebellum at the surface of the cerebellar fossa, which potentially may produce slight variation in fossa volume measurements. Possibly more problematic is that the distal extent of the neural flocculus is impossible to determine with certainty in most Type 5 fossae (the most commonly encountered type), and a potentially significant proportion of volume in these may relate to vascular space rather than neural tissue. An extensive survey of vascular versus neural occupancy of the floccular fossa in living bird species is needed to test the nature and extent of this variability. Until floccular fossa vascularity is better known in extant birds, informed estimates of neural volume in the floccular fossae of fossil birds cannot be made. Similarly, estimates of flying ability based on the apparently large flocculi observed on the endocranial casts of non-avian dinosaurs [12], [13] and pterosaurs [9] should be regarded with additional caution, especially as some of these taxa are phylogenetically distant from extant birds and may have had novel neural or vascular structures that are unknown in modern birds.

Because maneuverability increases as BI values decrease [39], one prediction is that species with low BI values should have larger flocculi. However, in this dataset the opposite is true. One possible reason for this is that larger birds tend to have larger brains and larger BI values, and the strong positive correlation between FFV and BCEVr volumes indicates they also have larger relative FFV values. The presence of large flying and flightless species in the dataset could potentially cause this positive relationship. However, although not shown, the removal of all taxa greater than 10 kg in the dataset (*Struthio*, *Dromaius*, *Casuarius*, *Rhea*, *Pezophaps*, *Cygnus* and *Vultur*: see Data S1) had no significant effect on this positive correlation between BI and relative FFV.

Larger flying birds are generally thermal or dynamic soarers (e.g., *Aquila*, *Buteo*, *Vultur*, *Diomedea*) that often spend long periods far from the ground, are incapable of tight maneuvering and experience relatively little in the way of powerful vertical accelerations through flapping flight [40], [41]. *Cygnus* is an exception in terms of engaging in extended periods of powerful flapping flight (but not agile aerial maneuvers), but the long neck of anseriforms serves to insulate their heads from the powerful vertical rise and fall during the wing beat cycle [45]. One explanation of the positive relationship between FFV and BI is that low VOR gains far from the ground might actually require a greater commitment of neural tissue to VOR processing, resulting in larger flocculi. If so, species that fly closer to the ground may not require such large flocculi because VOR gain from the optic flow field is greater. A factor potentially weakening this relationship might be that major differences in VOR gain between flying far from the ground and close to the ground, as well as changes in VOR gain during landing maneuvers and subsequent terrestrial locomotion, require a particularly large degree of plasticity and adaptation. If this extra capacity requires increases in neural tissue it could affect any prediction of how flocculus volume will vary based on basic quantification of ‘normal’ flight in a given taxon. The reasons for this positive relationship are therefore potentially complex, and the relatively weak relationship (r^2^ of ca. 0.1) should be regarded with caution until more is known about how flocculus structure and function differs among taxa.

It is also noteworthy that flightless birds have relative FFV values in the upper range of the dataset, and that there is a high degree of overlap with flying species. Since these taxa do not experience the diversity of flight-based optic flow environments mentioned above, the flocculus might be expected to decrease in relative size during the evolution of flightlessness if the role of the flocculus in processing the VCR and VOR is so important for flight. This size decrease has clearly not occurred, so assuming vascular structures in the floccular fossa of these taxa are not significantly larger than in volant species, the region must remain important in flightless species for other reasons. The high FFV values of flightless taxa might simply represent retention of the condition seen in their volant ancestors and represent examples of phylogenetic conservatism [20], or represent exaptation of functions for ground-based bipedal locomotion from those once used for flight. For instance, the VOR is important for visual field processing in other locomotory modes such as running and wing- and foot-propelled diving, and together with the VCR the flocculus must play a crucial role in maintaining and changing posture [46], [47].

The strong correlation between FFV and BCEVr indicates that changes in the region’s size must generally keep pace with those of overall brain size during evolution, even though overall size changes may be a result of mosaic rather than concerted regional size change [48]. However, the apparent expansion of the flocculus may not result solely from increases in floccular tissue, and other parts of the vestibulocerebellum may also be involved. For instance, the uvula and nodulus lie medial to the floccular lobes and integrate optic flow and vestibular information to process postural and locomotor reflexes relating to head translation and stabilization [49]. Consequently, uvula-nodulus processing must be important for flight, and is probably also relevant to debates [50] about the nature and purpose of avian ‘head bobbing’ during terrestrial locomotion. The uvula-nodulus might be expected to be larger in volant taxa, although this enlargement would not be obvious in an endocranial cast. However, expansion of the uvula-nodulus could conceivably have led to extrusion of the flocculus into the loop of the rostral semicircular canal. If so, the apparently large flocculi of birds may actually be an expression of a functionally enlarged flocculus-uvula-nodulus complex. A comparison of vestibulocerebellar size, the combination of the uvula and nodulus, across extant birds suggests that the uvula-nodulus is smaller in ‘strong fliers’ [51], which were a collection of species defined by Larsell [52] as species that fly long distances or are highly maneuverable and included a diverse array of taxa (waterfowl, swifts, raptors, hummingbirds, seabirds, terns, penguins and swallows). A more accurate categorization of flight behavior as well as a survey of floccular volumes would provide some insight into whether uvula-nodulus expansion is related to floccular expansion and the relationship that both have to flight behavior, if any.

Enhanced visual stabilization has been suggested to be important for stable terrestrial bipedal locomotion [53], and the possibility exists that the enlarged and protruding flocculus (possibly related to uvula-nodulus expansion) seen in birds, non-avian theropod dinosaurs and pterosaurs (but not extant crocodiles) actually relates to the evolution of bipedal terrestrial locomotion in Archosauria. Compared with quadrupedal locomotion, bipedality is inherently unstable and requires enhanced control through vestibular and proprioceptive feedback [53], [54]. As such, primary enlargement of the flocculus/uvula-nodulus to cope with these demands may have occurred in the common ancestor of dinosaurs, birds and pterosaurs, or have arisen multiple times if bipedality evolved separately in several archosaur clades. The flocculus is not laterally expanded in squamates [55], but there is some lateral expansion to form a discernible floccular ‘lobe’ in chelonians and *Alligator* [56]. However, no volumetric measurements are available for the flocculus or uvula-nodulus in any of these taxa and nothing is known about the hodological or physiological organization of the flocculus and associated vestibulocerebellum in squamates, turtles or crocodilians [20]. Testing whether the evolution of bipedality is indeed associated with an expansion of the vestibulocerebellum (indexed by expansion of the flocculus) may be possible by amassing data for a broader range of species. For example, µCT analyses of key basal archosaur taxa and crocodile-line archosaur (pseudosuchian) taxa, including both obligate quadrupeds and rare bipeds (such as *Effigia*), would represent an important step toward testing the hypothesis that the multiple instances of flight evolution in Archosauria were aided by the possession of a basic ‘flying brain’ in a common archosaur ancestor [19].

There is a degree of within-clade variation in floccular fossa type, but some clades (notably Palaeognathae – Type 3; Psittaciformes – Type 1) appear to possess one type only. Across clades that exhibit variation, there is some evidence that some fossa types may be more common among taxa exhibiting broad differences in locomotor behavior. For instance, Type 4 fossae are present in *Apus*, *Coracias*, *Corvus*, *Creagrus*, *Falco*, *Hirundo*, *Larus*, *Phaethon*, *Podargus*, *Trogon* and *Tyrannus*. These taxa represent at least eight different avian orders, but all species exhibit good and sometimes exceptional maneuverability. These short and dome-like fossae contrast strongly with the long and broad Type 3 fossae found in the flightless palaeognaths, but also in the weak flier *Rhynchotus* and the soaring *Diomedea*. Further work is needed to determine whether floccular fossa morphology may be more useful than size for inferring flying ability.

Given the high FFV values for extant flightless birds, the apparently large flocculi seen in some bird-like theropod dinosaurs mentioned above might support the hypothesis that these taxa are secondarily flightless birds. This suggestion is enhanced by the morphology of most (e.g., [14], [57], [58]), but not all (e.g., possibly not *Incisivosaurus* [13]), known theropod flocculi, which most closely approximate the Type 3 fossae of palaeognaths described here. However, until a more comprehensive survey of fossa morphology can be undertaken, the possibility that the Type 3 fossa represents a plesiomorphic morphology that arose in non-avian theropods and that was retained by birds cannot be discounted. Consequently, the results of our numerical analyses and morphological investigations neither support nor refute derivation of these taxa from volant ancestors. However, overwhelming anatomical evidence from all other parts of the skeleton in these bird-like taxa strongly indicates that they genuinely are non-avian dinosaurs.

Our results provide a reminder of the limitations of the brain cavity as a source of neural information. A growing number of quantitative investigations of relative brain region volume using wet specimen datasets are providing useful behavioral characterizations of brain composition and shape [59], [60]. By comparison, few studies [61] have attempted empirical investigation of brain cavity morphology for inferring behavior. Despite the advent of X-ray µCT, the field of avian paleoneurology will remain limited to qualitative assessment of brain shape or quantitative assessment of overall brain size until such studies are performed.

## Supporting Information

Data S1 **Measurements and flight-style coding for all included avian taxa.**
(XLS)

Scanning S1 **Scanning parameters, specimen numbers and name of who scanned and segmented each specimen.**
(PDF)
