# Supplementary material for: Avian Cerebellar Floccular Fossa Size Is Not a Proxy for Flying Ability in Birds
Source: PLoS One. 2013 Jun 25;8(6):e67176. doi: 10.1371/journal.pone.0067176 (PMC3692442; doi:10.1371/journal.pone.0067176)
Supplement: Scanning S1 — Scanning parameters, specimen numbers and name of who scanned and segmented each specimen. (PDF) [file pone.0067176.s002.pdf]

Scan and Segmentation Details

Note: All image stacks were down-sampled from the original 16-bit depth to 8-bit, and cropped to region of interest from the original image stack to reduce processing requirements. Voxel size reduction was performed where indicated to further reduce processing where image stacks remained too large to be easily manipulated after cropping and bit depth reduction.

Institutional abbreviations: NHMUK, The Natural History Museum; NMS, National Museums Scotland.

Acanthorhynchus superciliosus (Western spinebill) NHMUK S/1966.51.209.
Scanned at The Natural History Museum, UK, by Richard Abel, using a Nikon Metrology HMX ST CT. Specimen scanned along axial axis as part of NERC Small Grant NE/H012176/1 awarded to Walsh, originally reconstructed as a 16-bit VGStudio Max VOL file and output from VGStudio Max 2.0 as an 8-bit BMP stack. Original resolution, 10 µm (1506 x 1211 pixel grid), final image stack resolution: 30 μm^3, 598 slices (764 x 940 pixel grid), field of view 22.92 mm. Endocranial cast segmention: Stig Walsh, flocculus cast removal: Stig Walsh.

Alca torda (Razorbill) NMS Z.2000.08.105.
Scanned at The University of Abertay, UK, by Stig Walsh, using a Nikon Metrology HMX ST CT. Specimen scanned along axial axis as part of NERC Small Grant NE/H012176/1 awarded to Walsh, originally reconstructed as a 16-bit VGStudio Max VOL file and output from VGStudio Max 2.0 as an 8-bit BMP stack. Resolution: 69 μm^3, 472 slices (408 x 438 pixel grid), field of view 28.08 mm. Endocranial cast segmention: Stig Walsh, flocculus cast removal: Stig Walsh.

Alcedo atthis (Common kingfisher) NMS collections (unregistered).
Scanned at The University of Abertay, UK, by Stig Walsh, using a Nikon Metrology HMX ST CT. Specimen scanned along axial axis as part of NERC Small Grant NE/H012176/1 awarded to Walsh, originally reconstructed as a 16-bit VGStudio Max VOL file and output from VGStudio Max 2.0 as an 8-bit BMP stack. Resolution: 32 μm^3, 598 slices (571 x 637 pixel grid), field of view 18.05 mm. Endocranial cast segmention: Stig Walsh, flocculus cast removal: Stig Walsh.

Amazona aestiva (Blue fronted amazon) NHMUK Palaeontology Collection 99.
Scanned at The Natural History Museum, UK, by Estelle Bourdon, using a Nikon Metrology HMX ST CT. Specimen scanned along axial axis as part of Intra-European Marie Curie Fellowship 255039, originally reconstructed as a 16-bit VGStudio Max 2.0 VOL file and output as a 16-bit DCM stack, then downsampled in Mimics 14.11 as an 8-bit MCS file. Original resolution, 29 µm (1379 x 1135 pixel grid), final image stack resolution: 50 μm^3, 766 slices (678 x 515 pixel grid), field of view 30.9 mm. Endocranial cast segmention: Stig Walsh, flocculus cast removal: Stig Walsh.

Apteryx haastii (Great spotted kiwi) NMS Z.1913.13.748.
Scanned at The University of Abertay, UK, by Stig Walsh, using a Nikon Metrology HMX ST CT. Specimen scanned along axial axis as part of NERC Small Grant NE/H012176/1 awarded to Walsh, originally reconstructed as a 16-bit VGStudio Max VOL file and output from VGStudio Max 2.0 as an 8-bit BMP stack. Resolution: 88 μm^3, 745 slices (429 x 464 pixel grid), field of view 37.77 mm. Endocranial cast segmention: Stig Walsh, flocculus cast removal: Stig Walsh.

Apus apus (Common swift) NMS Z.1931.43.
Scanned at The University of Abertay, UK, by Stig Walsh, using a Nikon Metrology HMX ST CT. Specimen scanned along axial axis as part of NERC Small Grant NE/H012176/1 awarded to Walsh, originally reconstructed as a 16-bit VGStudio Max VOL file and output from VGStudio Max 2.0 as an 8-bit BMP stack. Resolution: 38 μm^3, 483 slices (478 x 473 pixel grid), field of view 18.18 mm. Endocranial cast segmention: Monja Knoll, flocculus cast removal: Stig Walsh.

Aquila chrysaetos (Golden eagle) NMS Z.1997.29.
Scanned at The University of Abertay, UK, by Stig Walsh, using a Nikon Metrology HMX ST CT. Specimen scanned along axial axis as part of NERC Small Grant NE/H012176/1 awarded to Walsh, originally reconstructed as a 16-bit VGStudio Max VOL file and output from VGStudio Max 2.0 as an 8-bit BMP stack. Resolution: 55 μm^3, 890 slices (914 x 893 pixel grid), field of view 50.13 mm. Endocranial cast segmention: Monja Knoll, flocculus cast removal: Stig Walsh.

Ara macao (Scarlet macaw) NMS Z.1931.43.
Scanned at The University of Abertay, UK, by Stig Walsh, using a Nikon Metrology HMX ST CT. Specimen scanned along axial axis as part of NERC Small Grant NE/H012176/1 awarded to Walsh, originally reconstructed as a 16-bit VGStudio Max VOL file and output from VGStudio Max 2.0 as an 8-bit BMP stack. Resolution: 65 μm^3, 716 slices (777 x 796 pixel grid), field of view 50.62 mm. Endocranial cast segmention: Stig Walsh, flocculus cast removal: Stig Walsh.

Ardea cineria (Grey heron) NMS Z.2002.64.
Scanned at The University of Abertay, UK, by Stig Walsh, using a Nikon Metrology HMX ST CT. Specimen scanned along axial axis as part of NERC Small Grant NE/H012176/1 awarded to Walsh, originally reconstructed as a 16-bit VGStudio Max VOL file and output from VGStudio Max 2.0 as an 8-bit BMP stack. Resolution: 88 μm^3, 529 slices (417 x 427 pixel grid), field of view 28.7 mm. Endocranial cast segmention: Stig Walsh, flocculus cast removal: Stig Walsh.

Aythya fuligula (Tufted duck) NHMUK S/1987.27.1.
Scanned at Nikon Metrology, Tring, UK, using a Nikon Metrology HMX ST CT. Specimen scanned along axial axis as part of NERC Small Grant NE/E008380/1 awarded to Barrett and Milner, originally reconstructed as a 16-bit VGStudio Max VOL file and output as a 16-bit DCM stack, then downsampled in Mimics 14.11 as an 8-bit MCS file. Final image stack resolution: 36 μm^3, 945 slices (940 x 1026 pixel grid), field of view 33.93 mm. Endocranial cast segmention: Stig Walsh, flocculus cast removal: Stig Walsh.

Buteo buteo (Common buzzard) NHMUK S/2007.139.12.
Scanned at Nikon Metrology, Tring, UK, using a Nikon Metrology HMX ST CT. Specimen scanned along axial axis as part of NERC Small Grant NE/E008380/1 awarded to Barrett and Milner, originally reconstructed as a 16-bit VGStudio Max VOL file and output as a 16-bit DCM stack, then downsampled in Mimics 14.11 as an 8-bit MCS file. Final image stack resolution 42 μm^3, 1019 slices (815 x 1112 pixel grid), field of view 34.22 mm. Endocranial cast segmention: Stig Walsh, flocculus cast removal: Stig Walsh.

Casuarius casuarius (Cassowary) NHMUK S/1939.12.9.964.
Scanned at Nikon Metrology, Tring, UK, using a Nikon Metrology HMX ST CT. Specimen scanned along axial axis as part of NERC Small Grant NE/E008380/1 awarded to Barrett and Milner, originally reconstructed as a 16-bit VGStudio Max VOL file and output as a 16-bit DCM stack, then downsampled in Mimics 14.11 as an 8-bit MCS file. Final image stack resolution: 149 μm^3, 1470 slices (654 x 650 pixel grid), field of view 97.19 mm. Endocranial cast segmention: Stig Walsh, flocculus cast removal: Stig Walsh.

Ciconia ciconia (White Stork) NHMUK 1859.9.6.393.
Scanned at Nikon Metrology, Tring, UK, using a Nikon Metrology HMX ST CT. Specimen scanned along axial axis as part of NERC Small Grant NE/E008380/1 awarded to Barrett and Milner, originally reconstructed as a 16-bit VGStudio Max VOL file and output as a 16-bit DCM stack, then downsampled in Mimics 14.11 as an 8-bit MCS file. Original resolution: 45 µm (1164 x 1232 pixel grid), final image stack resolution: 70 μm^3, 506 slices (562 x 568 pixel grid), field of view 39.34 mm. Endocranial cast segmention: Stig Walsh, flocculus cast removal: Stig Walsh.

Circus cyaneus (Hen harrier) NMS Z.1995.188.
Scanned at The University of Abertay, UK, by Stig Walsh, using a Nikon Metrology HMX ST CT. Specimen scanned along axial axis as part of NERC Small Grant NE/H012176/1 awarded to Walsh, originally reconstructed as a 16-bit VGStudio Max VOL file and output as an 8-bit BMP stack. Original resolution, 36 µm (1558 x 1576 pixel grid), final image stack resolution: 55 μm^3, 709 slices (903 x 514 pixel grid), field of view 50.05 mm. Endocranial cast segmention: Stig Walsh, flocculus cast removal: Stig Walsh.

Columba livia (Rock dove) NMS collections (unregistered).
Scanned at The University of Abertay, UK, by Stig Walsh, using a Nikon Metrology HMX ST CT. Specimen scanned along axial axis as part of NERC Small Grant NE/H012176/1 awarded to Walsh, originally reconstructed as a 16-bit VGStudio Max VOL file and output from VGStudio Max 2.0 as an 8-bit BMP stack. Resolution: 56 μm^3, 1018 slices (453 x 452 pixel grid), field of view 25.56 mm. Endocranial cast segmention: Stig Walsh, flocculus cast removal: Stig Walsh.

Coracias garrulous (European roller) NMS Z.1994.44.
Scanned at The University of Abertay, UK, by Stig Walsh, using a Nikon Metrology HMX ST CT. Specimen scanned along axial axis as part of NERC Small Grant NE/H012176/1 awarded to Walsh, originally reconstructed as a 16-bit VGStudio Max VOL file and output from VGStudio Max 2.0 as an 8-bit BMP stack. Resolution: 44 μm^3, 477 slices (504 x 546 pixel grid), field of view 22.17 mm. Endocranial cast segmention: Stig Walsh, flocculus cast removal: Stig Walsh.

Corvus corax (Raven) NHMUK S/1979.66.160.
Scanned at Nikon Metrology, Tring, UK, using a Nikon Metrology HMX ST CT. Specimen scanned along axial axis as part of NERC Small Grant NE/E008380/1 awarded to Barrett and Milner, originally reconstructed as a 16-bit VGStudio Max VOL file and output as a 16-bit DCM stack, then downsampled in Mimics 14.11 as an 8-bit MCS file. Resolution: 43 μm^3, 1380 slices (1107 x 1273 pixel grid), field of view 47.6 mm. Endocranial cast segmention: Stig Walsh, flocculus cast removal: Stig Walsh.

Creagrus furcatus (Swallow tailed gull) NHMUK S/1967.19.6.
Scanned at The Natural History Museum, UK, by Richard Abel, using a Nikon Metrology HMX ST CT. Specimen scanned along axial axis as part of NERC Small Grant NE/H012176/1 awarded to Walsh, originally reconstructed as a 16-bit VGStudio Max VOL file and output from VGStudio Max 2.0 as an 8-bit BMP stack. Resolution: 43 μm^3, 1034 slices (1412 x 1467 pixel grid), field of view 40.96 mm. Endocranial cast segmention: Stig Walsh, flocculus cast removal: Stig Walsh.

Cygnus olor (Mute swan) NHMUK S/1995.11.1.
Scanned at Nikon Metrology, Tring, UK, using a Nikon Metrology HMX ST CT. Specimen scanned along axial axis as part of NERC Small Grant NE/E008380/1 awarded to Barrett and Milner, originally reconstructed as a 16-bit VGStudio Max VOL file and output as a 16-bit DCM stack, then downsampled in Mimics 14.11 as an 8-bit MCS file. Resolution: 50 μm^3, 1048 slices (832 x 1014 pixel grid), field of view 41.68 mm. Endocranial cast segmention: Stig Walsh, flocculus cast removal: Stig Walsh.

Diomedea exulans (Wandering albatross) NMS Z.1921.143.1630.
Scanned at The University of Abertay, UK, by Stig Walsh, using a Nikon Metrology HMX ST CT. Specimen scanned along axial axis as part of NERC Small Grant NE/H012176/1 awarded to Walsh, originally reconstructed as a 16-bit VGStudio Max VOL file and output from VGStudio Max 2.0 as an 8-bit BMP stack. Resolution: 73 μm^3, 965 slices (998 x 818 pixel grid), field of view 72.61 mm. Endocranial cast segmention: Stig Walsh, flocculus cast removal: Stig Walsh.

Dromaius novaehollandiae (Emu) NHMUK S/2001.50.1.
Scanned at Nikon Metrology, Tring, UK, using a Nikon Metrology HMX ST CT. Specimen scanned along axial axis as part of NERC Small Grant NE/E008380/1 awarded to Barrett and Milner, originally reconstructed as a 16-bit VGStudio Max VOL file and output as a 16-bit DCM stack, then downsampled in Mimics 14.11 as an 8-bit MCS file. Resolution: 93 μm^3, 798 slices (883 x 810 pixel grid), field of view 82.17 mm. Endocranial cast segmention: Stig Walsh, flocculus cast removal: Stig Walsh.

Eudyptula sp. (Little penguin) NHMUK A3915.
Scanned at The Natural History Museum, UK by Estelle Bourdon, using a Nikon Metrology HMX ST CT. Specimen scanned along axial axis as part of Intra-European Marie Curie Fellowship 255039, originally reconstructed as a 16-bit VGStudio Max VOL file and routput as a 16-bit DCM stack, then downsampled in Mimics 14.11 as an 8-bit MCS file. Original resolution, 27 µm (1566 x 1215 pixel grid), final image stack resolution: 50 μm^3, 836 slices (678 x 632 pixel grid), field of view 33.9 mm. Endocranial cast segmention: Stig Walsh, flocculus cast removal: Stig Walsh.

Falco subbuteo (Eurasian hobby) NHMUK S/1974.16.1.
Scanned at The Natural History Museum, UK by Richard Abel, using a Nikon Metrology HMX ST CT. Specimen scanned along axial axis as part of NERC Small Grant NE/H012176/1 awarded to Walsh, originally reconstructed as a 16-bit VGStudio Max VOL file and output from VGStudio Max 2.0 as an 8-bit BMP stack. Resolution: 32 μm^3, 797 slices (1046 x 943 pixel grid), field of view 33.25 mm. Endocranial cast segmention: Stig Walsh, flocculus cast removal: Stig Walsh.

Falco tinnunculus (Common kestrel) NMS collections (unregistered).
Scanned at The University of Abertay, UK, by Stig Walsh, using a Nikon Metrology HMX ST CT. Specimen scanned along axial axis as part of NERC Small Grant NE/H012176/1 awarded to Walsh,

originally reconstructed as a 16-bit VGStudio Max VOL file and output from VGStudio Max 2.0 as an 8-bit BMP stack. Resolution: 44 μm^3, 648 slices (704 x 588 pixel grid), field of view 30.96 mm. Endocranial cast segmention: Stig Walsh, flocculus cast removal: Stig Walsh.

Fregata magnificens (Magnificent frigate bird) NMS Z.2000.111.2.
Scanned at The University of Abertay, UK, by Stig Walsh, using a Nikon Metrology HMX ST CT. Specimen scanned along axial axis as part of NERC Small Grant NE/H012176/1 awarded to Walsh, originally reconstructed as a 16-bit VGStudio Max VOL file and output from VGStudio Max 2.0 as an 8-bit BMP stack. Resolution: 41 μm^3, 1161 slices (873 x 793 pixel grid), field of view 35.5 mm. Endocranial cast segmention: Monja Knoll, flocculus cast removal: Stig Walsh.

Fulmarus glacialis (Northern fulmar) NMS Z.2003.181.2.
Scanned at The University of Abertay, UK, by Stig Walsh, using a Nikon Metrology HMX ST CT. Specimen scanned along axial axis as part of NERC Small Grant NE/H012176/1 awarded to Walsh, originally reconstructed as a 16-bit VGStudio Max VOL file and output from VGStudio Max 2.0 as an 8-bit BMP stack. Resolution: 74 μm^3, 752 slices (641 x 610 pixel grid), field of view 32.05 mm. Endocranial cast segmention: Stig Walsh, flocculus cast removal: Stig Walsh.

Gallus gallus (Red junglefowl) NMS Z.1931.43.
Scanned at The University of Abertay, UK, by Stig Walsh, using a Nikon Metrology HMX ST CT. Specimen scanned along axial axis as part of NERC Small Grant NE/H012176/1 awarded to Walsh, originally reconstructed as a 16-bit VGStudio Max VOL file and output from VGStudio Max 2.0 as an 8-bit BMP stack. Resolution: 55 μm^3, 1534 slices (716 x 685 pixel grid), field of view 39.7 mm. Endocranial cast segmention: Stig Walsh, flocculus cast removal: Stig Walsh.

Gavia immer (Great northern loon) NMS collections (unregistered).
Scanned at The University of Abertay, UK, by Stig Walsh, using a Nikon Metrology HMX ST CT. Specimen scanned along axial axis as part of NERC Small Grant NE/H012176/1 awarded to Walsh, originally reconstructed as a 16-bit VGStudio Max VOL file and output from VGStudio Max 2.0 as an 8-bit BMP stack. Resolution: 81 μm^3, 699 slices (469 x 458 pixel grid), field of view 38.5 mm. Endocranial cast segmention: Stig Walsh, flocculus cast removal: Stig Walsh.

Gelochelidon nilotica (Gull billed tern) NMS collections (unregistered; cat. no. 613, no. 373).
Scanned at The University of Abertay, UK, by Stig Walsh, using a Nikon Metrology HMX ST CT. Specimen scanned along axial axis as part of NERC Small Grant NE/H012176/1 awarded to Walsh, originally reconstructed as a 16-bit VGStudio Max VOL file and output from VGStudio Max 2.0 as an 8-bit BMP stack. Resolution: 63 μm^3, 486 slices (491 x 401 pixel grid), field of view 30.97 mm. Endocranial cast segmention: Stig Walsh, flocculus cast removal: Stig Walsh.

Grus grus (Common crane) NMS Z.1904.80.6.
Scanned at The University of Abertay, UK, by Stig Walsh, using a Nikon Metrology HMX ST CT. Specimen scanned along axial axis as part of NERC Small Grant NE/H012176/1 awarded to Walsh, originally reconstructed as a 16-bit VGStudio Max VOL file and output from VGStudio Max 2.0 as an 8-bit BMP stack. Resolution: 54 μm^3, 980 slices (428 x 451 pixel grid), field of view 50.2 mm. Endocranial cast segmention: Stig Walsh, flocculus cast removal: Stig Walsh.

Hirundo rustica (Barn swallow) NMS collections (unregistered).
Scanned at The University of Abertay, UK, by Stig Walsh, using a Nikon Metrology HMX ST CT. Specimen scanned along axial axis as part of NERC Small Grant NE/H012176/1 awarded to Walsh, originally reconstructed as a 16-bit VGStudio Max VOL file and output from VGStudio Max 2.0 as an 8-bit BMP stack. Resolution: 38 μm^3, 450 slices (357 x 372 pixel grid), field of view 13.52 mm. Endocranial cast segmention: Stig Walsh, flocculus cast removal: Stig Walsh.

Larus argentatus (Herring gull) NMS collections (unregistered).
Scanned at The University of Abertay, UK, by Stig Walsh, using a Nikon Metrology HMX ST CT. Specimen scanned along axial axis as part of NERC Small Grant NE/H012176/1 awarded to Walsh, originally reconstructed as a 16-bit VGStudio Max VOL file and output from VGStudio Max 2.0 as an 8-bit BMP stack. Resolution: 85 μm^3, 499 slices (388 x 486 pixel grid), field of view 32.84 mm. Endocranial cast segmention: Stig Walsh, flocculus cast removal: Stig Walsh.

Opisthocomus hoatzin (Hoatzin) NHMUK S/1892.7.11.1.
Scanned at The Natural History Museum, UK, by Richard Abel, using a Nikon Metrology HMX ST CT. Specimen scanned along axial axis as part of NERC Small Grant NE/H012176/1 awarded to Walsh, originally reconstructed as a 16-bit VGStudio Max VOL file and output from VGStudio Max 2.0 as an 8-bit BMP stack. Resolution: 32 μm^3, 923 slices (829 x 984 pixel grid), field of view 26.36 mm. Endocranial cast segmention: Stig Walsh, flocculus cast removal: Stig Walsh.

Pandion haliaetus (Osprey) NMS Z.1995.35.
Scanned at The University of Abertay, UK, by Stig Walsh, using a Nikon Metrology HMX ST CT. Specimen scanned along axial axis as part of NERC Small Grant NE/H012176/1 awarded to Walsh, originally reconstructed as a 16-bit VGStudio Max VOL file and output from VGStudio Max 2.0 as an 8-bit BMP stack. Resolution: 69 μm^3, 587 slices (683 x 566 pixel grid), field of view 47.01 mm. Endocranial cast segmention: Stig Walsh, flocculus cast removal: Stig Walsh.

Pelagodroma marina (White faced storm petrel) NMS Z.1898.30 (cat. no. 294).
Scanned at The University of Abertay, UK, by Stig Walsh, using a Nikon Metrology HMX ST CT. Specimen scanned along axial axis as part of NERC Small Grant NE/H012176/1 awarded to Walsh, originally reconstructed as a 16-bit VGStudio Max VOL file and output from VGStudio Max 2.0 as an 8-bit BMP stack. Resolution: 36 μm^3, 550 slices (402 x 408 pixel grid), field of view 14.55 mm. Endocranial cast segmention: Stig Walsh, flocculus cast removal: Stig Walsh.

Pelecanoides urinatrix (Common diving petrel) NHMUK A4299.
Scanned at The Natural History Museum, UK, by Estelle Bourdon, using a Nikon Metrology HMX ST CT. Specimen scanned along axial axis as part of Intra-European Marie Curie Fellowship 255039, originally reconstructed as a 16-bit VGStudio Max VOL file and output as a 16-bit DCM stack, then downsampled in Mimics 14.11 as an 8-bit MCS file. Original resolution, 16 µm (1293 x 1124 pixel grid), final image stack resolution: 50 μm^3, 444 slices (360 x 305 pixel grid), field of view 18.0 mm. Endocranial cast segmention: Stig Walsh, flocculus cast removal: Stig Walsh.

Pelecanus erythrorhynchus (American white pelican) NHMUK S/1969.3.1.
Scanned at The Natural History Museum, UK, by Estelle Bourdon, using a Nikon Metrology HMX ST CT. Specimen scanned along axial axis as part of Intra-European Marie Curie Fellowship 255039,

originally reconstructed as a 16-bit VGStudio Max VOL file and output as a 16-bit DCM stack, then downsampled in Mimics 14.11 as an 8-bit MCS file. Resolution: 49 μm^3, 1285 slices (800 x 687 pixel grid), field of view 38.96mm. Endocranial cast segmention: Stig Walsh, flocculus cast removal: Stig Walsh.

Pezophaps solitaria (Solitaire) NHMUK A1376.
Scanned at The Natural History Museum, UK, by Estelle Bourdon, using a Nikon Metrology HMX ST CT. Specimen scanned along axial axis as part of Intra-European Marie Curie Fellowship 255039, originally reconstructed as a 16-bit VGStudio Max VOL file and output as a 16-bit DCM stack, then downsampled in Mimics 14.11 as an 8-bit MCS file. Resolution: 47 μm^3, 1208 slices (683 x 719 pixel grid), field of view 31.95 mm. Endocranial cast segmention: Stig Walsh, flocculus cast removal: Stig Walsh.

Phaethon lepturus (White tailed tropic bird) NHMUK 1884.2.29.10.
Scanned at The Natural History Museum, UK, by Richard Abel, using a Nikon Metrology HMX ST CT. Specimen scanned along axial axis as part of NERC Small Grant NE/H012176/1 awarded to Walsh, originally reconstructed as a 16-bit VGStudio Max VOL file and output from VGStudio Max 2.0 as an 8-bit BMP stack. Original resolution, 37 µm (1393 x 1261 pixel grid), final image stack resolution: 74 μm^3, 424 slices (439 x 440 pixel grid), field of view 32.55 mm. Endocranial cast segmention: Stig Walsh, flocculus cast removal: Stig Walsh.

Phalacrocorax carbo (Great cormorant) NMS Z.2000.27.04.
Scanned at The University of Abertay, UK, by Stig Walsh, using a Nikon Metrology HMX ST CT. Specimen scanned along axial axis as part of NERC Small Grant NE/H012176/1 awarded to Walsh, originally reconstructed as a 16-bit VGStudio Max VOL file and output from VGStudio Max 2.0 as an 8-bit BMP stack. Resolution: 80 μm^3, 851 slices (496 x 483 pixel grid), field of view 39.74 mm. Endocranial cast segmention: Stig Walsh, flocculus cast removal: Stig Walsh.

Phalacrocorax harrisi (Galapagos cormorant) NHMUK S/1973.1.10.
Scanned at The Natural History Museum, UK, by Richard Abel, using a Nikon Metrology HMX ST CT. Specimen scanned along axial axis as part of NERC Small Grant NE/H012176/1 awarded to Walsh, originally reconstructed as a 16-bit VGStudio Max VOL file and output from VGStudio Max 2.0 as an 8-bit BMP stack. Resolution: 50 μm^3, 1306 slices (681 x 728 pixel grid), field of view 34.13 mm. Endocranial cast segmention: Stig Walsh, flocculus cast removal: Stig Walsh.

Phasianus colchicus (Common pheasant) NMS Z.1931.43.
Scanned at The University of Abertay, UK, by Stig Walsh, using a Nikon Metrology HMX ST CT. Specimen scanned along axial axis as part of NERC Small Grant NE/H012176/1 awarded to Walsh, originally reconstructed as a 16-bit VGStudio Max VOL file and output from VGStudio Max 2.0 as an 8-bit BMP stack. Resolution: 58 μm^3, 616 slices (486 x 521 pixel grid), field of view 28.19 mm. Endocranial cast segmention: Monja Knoll, flocculus cast removal: Stig Walsh.

Podargus strigoides (Tawny frogmouth) NHMUK S/1969.4.41.
Scanned at The Natural History Museum, UK, by Richard Abel, using a Nikon Metrology HMX ST CT. Specimen scanned along axial axis as part of NERC Small Grant NE/H012176/1 awarded to Walsh, originally reconstructed as a 16-bit VGStudio Max VOL file and output from VGStudio Max as an 8-bit

BMP stack. Resolution: 33 µm^3, 1003 slices (715 x 700 pixel grid), field of view 23.73 mm. Endocranial cast segmention: Stig Walsh, flocculus cast removal: Stig Walsh.

Podiceps cristatus (Great crested grebe) NMS Z.1990.74.
Scanned at The University of Abertay, UK, by Stig Walsh, using a Nikon Metrology HMX ST CT. Specimen scanned along axial axis as part of NERC Small Grant NE/H012176/1 awarded to Walsh, originally reconstructed as a 16-bit VGStudio Max VOL file and output from VGStudio Max 2.0 as an 8-bit BMP stack. Resolution: 75 µm^3, 635 slices (421 x 363 pixel grid), field of view 31.39 mm. Endocranial cast segmention: Stig Walsh, flocculus cast removal: Stig Walsh.

Ramphastos dicolorus (Toucan) NMS Z.1931.43.
Scanned at The University of Abertay, UK, by Stig Walsh, using a Nikon Metrology HMX ST CT. Specimen scanned along axial axis as part of NERC Small Grant NE/H012176/1 awarded to Walsh, originally reconstructed as a 16-bit VGStudio Max VOL file and output from VGStudio Max 2.0 as an 8-bit BMP stack. Resolution: 85 µm^3, 405 slices (375 x 383 pixel grid), field of view 31.74 mm. Endocranial cast segmention: Stig Walsh, flocculus cast removal: Stig Walsh.

Rhea Americana (Greater rhea) NHMUK S/1973.66.2.
Scanned at The Natural History Museum, UK, by Estelle Bourdon, using a Nikon Metrology HMX ST CT. Specimen scanned along axial axis as part of Intra-European Marie Curie Fellowship 255039, originally reconstructed as a 16-bit VGStudio Max VOL file and output as a 16-bit DCM stack then downsampled in Mimics 14.11 as an 8-bit MCS file. Resolution: 69 µm^3, 998 slices (662 x 734 pixel grid), field of view 45.59 mm. Endocranial cast segmention: Stig Walsh, flocculus cast removal: Stig Walsh.

Rhynchops niger (Black skimmer) NHMUK S/1973.62.2.
Scanned at The Natural History Museum, UK, by Estelle Bourdon, using a Nikon Metrology HMX ST CT. Specimen scanned along axial axis as part of Intra-European Marie Curie Fellowship 255039, originally reconstructed as a 16-bit VGStudio Max VOL file and output as a 16-bit DCM stack then downsampled in Mimics 14.11 as an 8-bit MCS file. Resolution: 24 µm^3, 1209 slices (1059 x 794 pixel grid), field of view 25.73 mm. Endocranial cast segmention: Stig Walsh, flocculus cast removal: Stig Walsh.

Rhynchotus ruficens (Red winged tinamou) NMS Z.1890.28.9.
Scanned at The University of Abertay, UK, by Stig Walsh, using a Nikon Metrology HMX ST CT. Specimen scanned along axial axis as part of NERC Small Grant NE/H012176/1 awarded to Walsh, originally reconstructed as a 16-bit VGStudio Max VOL file and output from VGStudio Max 2.0 as an 8-bit BMP stack. Resolution: 56 µm^3, 594 slices (574 x 471 pixel grid), field of view 32.38 mm. Endocranial cast segmention: Stig Walsh, flocculus cast removal: Stig Walsh.

Sagittarius serpentarius (Secretary bird) NMS Z.1931.120.7.
Scanned at The University of Abertay, UK, by Stig Walsh, using a Nikon Metrology HMX ST CT. Specimen scanned along axial axis as part of NERC Small Grant NE/H012176/1 awarded to Walsh, originally reconstructed as a 16-bit VGStudio Max VOL file and output from VGStudio Max 2.0 as an 8-bit BMP stack. Resolution: 58 µm^3, 912 slices (766 x 746 pixel grid), field of view 44.43 mm. Endocranial cast segmention: Stig Walsh, flocculus cast removal: Stig Walsh.

Selasphorus rufus (Rufus hummingbird) NMS Z.2002.108.2.
Scanned at The University of Abertay, UK, by Stig Walsh, using a Nikon Metrology HMX ST CT. Specimen scanned along axial axis as part of NERC Small Grant NE/H012176/1 awarded to Walsh, originally reconstructed as a 16-bit VGStudio Max VOL file and output from VGStudio Max 2.0 as an 8-bit BMP stack. Resolution: 12 μm^3, 654 slices (1794 x 1496 pixel grid), field of view 21.35 mm. Endocranial cast segmention: Stig Walsh, flocculus cast removal: Stig Walsh.

Steatornis caripensis (Oilbird) NHMUK S/1869.6.20.1.
Scanned at The Natural History Museum, UK, by Richard Abel, using a Nikon Metrology HMX ST CT. Specimen scanned along axial axis as part of NERC Small Grant NE/H012176/1 awarded to Walsh, originally reconstructed as a 16-bit VGStudio Max VOL file and output from VGStudio Max as an 8-bit BMP stack. Resolution: 35 μm^3, 947 slices (1093 x 711 pixel grid), field of view 38.57 mm. Endocranial cast segmention: Stig Walsh, flocculus cast removal: Stig Walsh.

Stercorarius skua (Skua) NHMUK Palaeontology Collection 71.
Scanned at The Natural History Museum, UK, by Estelle Bourdon, using a Nikon Metrology HMX ST CT. Specimen scanned along axial axis as part of Intra-European Marie Curie Fellowship 255039, originally reconstructed as a 16-bit VGStudio Max VOL file and output as a 16-bit DCM stack then downsampled in Mimics 14.11 as an 8-bit MCS file. Original resolution 38 µm (1556 x 1088 pixel grid), final image stack resolution: 50 μm^3, 881 slices (664 x 633 pixel grid), field of view 33.2 mm. Endocranial cast segmention: Stig Walsh, flocculus cast removal: Stig Walsh.

Strigops habroptila (Kakapo) NHMUK Palaeontology Collection 46663.
Scanned at The Natural History Museum, UK, by Estelle Bourdon, using a Nikon Metrology HMX ST CT. Specimen scanned along axial axis as part of Intra-European Marie Curie Fellowship 255039, originally reconstructed as a 16-bit VGStudio Max VOL file and output as a 16-bit DCM stack then downsampled in Mimics 14.11 as an 8-bit MCS file. Original resolution, 36 µm (1257 x 1273 pixel grid), final image stack resolution: 71 μm^3, 1997 slices (529 x 535 pixel grid), field of view 37.76 mm. Endocranial cast segmention: Stig Walsh, flocculus cast removal: Stig Walsh.

Struthio camelus (Ostrich) NHMUK S/1927.2.5.1.
Scanned at Nikon Metrology, Tring, UK, using a Nikon Metrology HMX ST CT. Specimen scanned along axial axis as part of NERC Small Grant NE/E008380/1 awarded to Barrett and Milner, originally reconstructed as a 16-bit VGStudio Max VOL file and output as a 16-bit DCM stack then downsampled in Mimics 14.11 as an 8-bit MCS file. Resolution: 137 μm^3, 774 slices (524 x 646 pixel grid), field of view 71.77 mm. Endocranial cast segmention: Stig Walsh, flocculus cast removal: Stig Walsh.

Tachyeres brachypterus (Falkland steamer duck) NHMUK S/2004.7.1.
Scanned at The Natural History Museum, UK, by Richard Abel, using a Nikon Metrology HMX ST CT. Specimen scanned along axial axis as part of NERC Small Grant NE/H012176/1 awarded to Walsh, originally reconstructed as a 16-bit VGStudio Max VOL file and output from VGStudio Max 2.0 as an 8-bit BMP stack. Resolution: 59 μm^3, 731 slices (567 x 606 pixel grid), field of view 33.66 mm. Endocranial cast segmention: Stig Walsh, flocculus cast removal: Stig Walsh.

Threskiornis aethiopicus (Sacred ibis) NHMUK S/1860.11.4.8.
Scanned at The Natural History Museum, UK, by Estelle Bourdon, using a Nikon Metrology HMX ST CT. Specimen scanned along axial axis as part of Intra-European Marie Curie Fellowship 255039, originally reconstructed as a 16-bit VGStudio Max VOL file, output as a 16-bit DCM stack the downsampled in Mimics 14.11 as an 8-bit MCS file. Original resolution, 30 µm (1211 x 1360 pixel grid), final image stack resolution: 50 μm^3, 889 slices (708 x 687 pixel grid), field of view 35.4 mm. Endocranial cast segmention: Stig Walsh, flocculus cast removal: Stig Walsh.

Trogon curucui (Blue crowned trogon) NHMUK S/1952.2.253.
Scanned at The Natural History Museum, UK, by Richard Abel, using a Nikon Metrology HMX ST CT. Specimen scanned along axial axis as part of NERC Small Grant NE/H012176/1 awarded to Walsh, originally reconstructed as a 16-bit VGStudio Max VOL file and output as an 8-bit BMP stack. Resolution: 22 μm^3, 726 slices (894 x 715 pixel grid), field of view 19.93 mm. Endocranial cast segmention: Stig Walsh, flocculus cast removal: Stig Walsh.

Tyrannus tyrannus (Eastern kingbird) NMS collections (unregistered, cat. 447, no. 33).
Scanned at The University of Abertay, UK, by Stig Walsh, using a Nikon Metrology HMX ST CT. Specimen scanned along axial axis as part of NERC Small Grant NE/H012176/1 awarded to Walsh, originally reconstructed as a 16-bit VGStudio Max VOL file output from VGStudio Max 2.0 as an 8-bit BMP stack. Resolution: 36 μm^3, 542 slices (510 x 481 pixel grid), field of view 18.46 mm. Endocranial cast segmention: Monja Knoll, flocculus cast removal: Stig Walsh.

Tyto alba (Barn owl) NHMUK S/1981.39.1.
Scanned at Nikon Metrology, Tring, UK, using a Nikon Metrology HMX ST CT. Specimen scanned along axial axis as part of NERC Small Grant NE/E008380/1 awarded to Barrett and Milner, originally reconstructed as a 16-bit VGStudio Max VOL file and output as a 16-bit DCM stack, then downsampled in Mimics 14.11 as an 8-bit MCS file. Resolution: 39 μm^3, 677 slices (811 x 1041 pixel grid), field of view 31.79 mm. Endocranial cast segmention: Stig Walsh, flocculus cast removal: Stig Walsh.

Vultur gryphus (Condor) NMS Z.1879.8.38.
Scanned at The University of Abertay, UK, by Stig Walsh, using a Nikon Metrology HMX ST CT. Specimen scanned along axial axis as part of NERC Small Grant NE/H012176/1 awarded to Walsh, originally reconstructed as a 16-bit VGStudio Max VOL file and output from VGStudio Max 2.0 as an 8-bit BMP stack. Resolution: 76 μm^3, 874 slices (786 x 812 pixel grid), field of view 59.42 mm. Endocranial cast segmention: Stig Walsh, flocculus cast removal: Stig Walsh.
